# Deep Learning-Based Football Player Detection in Videos

**DOI:** 10.1155/2022/3540642

**Published:** 2022-07-12

**Authors:** Tianyi Wang, Tongyan Li

**Affiliations:** College of Physical Education, Qiqihar University, Qiqihar 161000, China

## Abstract

The main task of football video analysis is to detect and track players. In this work, we propose a deep convolutional neural network-based football video analysis algorithm. This algorithm aims to detect the football player in real time. First, five convolution blocks were used to extract a feature map of football players with different spatial resolution. Then, features from different levels are combined together with weighted parameters to improve detection accuracy and adapt the model to input images with various resolutions and qualities. Moreover, this algorithm can be extended to a framework for detecting players in any other sports. The experimental results assure the effectiveness of our algorithm.

## 1. Introduction

In recent years, computer science has demonstrated great potential in the sport fields. For example, computer vision-based virtual reality was used for sports posture correction, [1] a computer vision-driven evaluation system was adopted for decision-making in sports training, [2] and object detection was used in sports analysis [3]. Sports analysis is crucial for improving athletes' performance. A classical method puts sensors to athletes' key positions and record raw data. Then data science methods are used to analyze the data and provide data-driven guidelines for training purposes [4]. However, additional sensors will increase the cost and may impair athletes' performance. Besides, one cannot ask his competitors to wear sensors in order to discover their strengths and weaknesses.

With the rapid development of computer vision technology, video analysis is more and more popular in helping improve athletes' expertise and training efficiency, and to prevent injuries [5]. This contactless technology enables coaches and athletes to train effectively, get rapid feedback, and minimize accidents. It can also help the coaches and athletes to analyze opponent teams' strength and weakness from past match videos, and design better strategies in future competitions [6].

The key steps for video analysis are player detection [7]. In this work, we develop a deep convolutional neural network-based football video analysis algorithm. First, videos were converted to sequential images, which are then passed through five consecutive convolutional layers with batch normalization and leaky ReLU as the activation function in order to extract features with different levels of spatial resolution. A residual connection around the first three convolutional blocks followed by normalization was used to take into account all levels of feature maps and improve the detection accuracy. The upsampled feature maps were combined with feature maps of lower levels to obtain a player confidence map and a corresponding player bounding box.

## 2. Related Work

Traditional model player detection includes connected component analysis [8], shallow convolutional neural networks [9], histogram of orientated gradients and support vector machines (HOG-SVM) [10], and deformable part model (DPM) [11].

**Figure 1:**
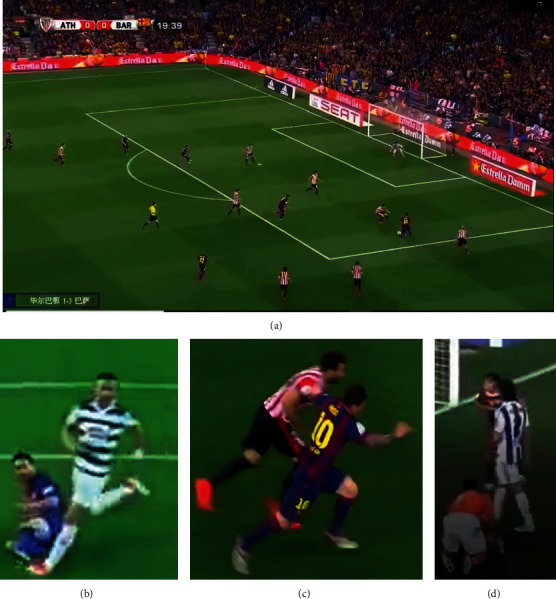
(a) A normal football match image for player detection. Difficult player detection tasks where players are nearby and (b) one player fell on the ground. (c) Plays' legs are overlapped. (d) One player is on his knees.

Figure 1 shows different situations in football player detection.  Figure 1(a) is a typical image where players are separated with each other. Traditional models generally can detect players in this situation, while they can hardly detect adjacent players (Figures 1(b)–1(d)) correctly in a harder situation. Besides, HOG-SVM needs domain knowledge and more labor work in order to conduct background segmentation [10]. Non-maximum suppression restricts the performance of DPM when detecting close players [12]. Other approaches such as motion or pixel or template-based methods have various restrictions, such as the player should not stand still, cannot wear a white jersey, shorts, and socks [13].

With the rapid development of computer vision, neural networks are dominating object detection algorithms [14, 15]. YOLO object detection algorithms have evolved from version 1 to version 5 with improving capabilities and performances which beat traditional algorithms, such as DPM [16–20]. However, YOLO is a large neural network with millions of parameters to train, which restricted its application in real-time object detection on portable devices.

One main challenge in player detection is background distraction. Complicated backgrounds make small objects harder to be detected. For example, if the background and players have similar colors, or players are too cluttered, it is hard to detect the players. Traditional methods use background subtraction approaches such as chromatic features [21], motion-based techniques [22], or median filtering [23] to preprocess the image frame.

Traditional deep neural networks suffer from vanishing gradients as the layers go deeper, leading to a worse performance than their shallow counterparts. He et al. reported using deep residual learning for image recognition [24], which overcome the vanishing gradient problem and enable training of very deep networks. The key part of the famous deep residual learning is the skip connection. The skip connection adds the output of the previous layer to the current layer and enables deep neural networks above 100 layers. The skip connection has been demonstrated to greatly enhance the model performance in image recognition.

In this work, we proposed a simple but efficient deep convolution neural network-based football player detection algorithm. It has two orders less parameters than YOLO and it can proceed an entire image in one pass. Besides, our model adopts a feature pyramid network design, which combines low level features with high level features. It helps differentiate an object with various sizes, and differentiate players with background clutter. A residual connection around the first three convolutional blocks followed by normalization was used to improve detection accuracy.

### 2.1. Model Architecture

**Figure 2:**
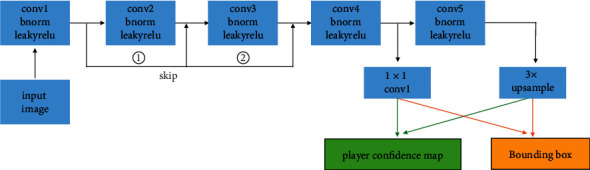
Model architecture of the football player detection system. First, an input image went through five consecutive convolutional layers with batch normalization and leaky ReLU as the activation function in order to extract features with different levels of spatial resolution. Then an upsampled feature map were combined with the feature level from the lower level to obtain the player confidence map and the corresponding player bounding box.

Figure 2 presents the model architecture of our football player detector. This architecture is built on a feature pyramid network (FPN) with residual connections around the first 3 convolution neural network blocks. FPN is well-known for its high accuracy and high speed in object detection. It has been demonstrated to perform better than both ResNet and Faster R-CNN in many object detection tasks. The advantages of ResNet are that it can be trained easily even with a large number of layers and it can avoid the vanishing gradient problem by using residual connections. Here, we combine the advantages of ResNet and FPN to build a new architecture which inherits both models' advantages. Another benefit of using residual connection on FPN is that it enable our detector to make the final detection based on all levels of feature maps instead of the last level of feature maps. Lower levels of feature maps give more spatial location information than higher levels of feature maps. Also, we use denser grids to improve detecting accuracy when two players are closed to each other. The probability of presence of a player inside a grid cell was encoded into the player confidence map, and the coordinates of the player was encoded in the bounding box. To find the player position in the confidence map, we apply non-maximum suppression to the player confidence map and then filter out all the locations above a threshold. Furthermore, we combine a high-level feature and a large receptive field to improve the detection accuracy on players with different gestures, such as players on knees and players who fell on the ground.

Table 1 gives the detailed information of our model. Filters are applied to generate feature maps. Their functions are to help extract various features from an image, such as edges, horizontal and vertical lines, and curves. Max pooling was applied here after filters in order to select the most significant features in the patch and ignore the average features. Our experiments shows that max pooling gives much better results than average pooling and min pooling. From conv1 to conv5, the extracted features changes from broad features to very specific features.

## 3. Experiments

### 3.1. Dataset

We use a public dataset, ISSIA-CNR soccer and soccer player detection datasets, to train and evaluate our model.

We use random football match video clips obtained from Tiktok to test the generality of our model.

### 3.2. Loss Function

The loss function in comprised of two parts, player classification loss and bounding box loss.

(1) $$\begin{array}{c} L_{p}=-\sum_{\left(x,y\right)\in \text{positive}}\log\;c_{x,y}-\sum_{\left(x,y\right)\in \text{negative}}\log\left(1-c_{x,y}\right), \end{array}$$

where *c*_*i*,*j*_ is the confidence score of the player at location (*x*, *y*).

As shown in (1), the player loss is binary cross entropy. Positive means the player exists in that position, negative means that position (*x*, *y*) does not have any player.

(2) $$\begin{array}{c} L_{b\text{box}}=\sum_{\left(x,y\right)\in \text{positive}}{\text{smooth}}_{L1}\left(l\left(x,y\right)-g\left(x,y\right)\right), \end{array}$$

where *l* (*x*, *y*) represents a predicted boxing box in position (*x*, *y*), and *g*(*x*, *y*) represents the corresponding ground truth (labeled) bounding box.

For the bounding box loss, we use similar smooth L1 loss as described in the f-CNN paper [25].

(3) $$\begin{array}{c} L=\frac{1}{N}\left(L_{p}+\beta L_{b\text{box}}\right), \end{array}$$

where *β* is a hyperparameter that decides the weight of bounding box loss in the totally loss.

### 3.3. Model Training

Both datasets, ISSIA-CNR soccer and soccer player detection datasets, were used for training. We adopted a stratified train test split to reduce bias. In other words, 80% ISSA-CNR soccer and 80% soccer player detection datasets were selected randomly as the training set, 20% ISSA-CNR soccer and 20% soccer player detection datasets as the test dataset. Furthermore, we conducted cross-validation in order to reduce overfitting and improve the model performance. AdamW was used as the optimizer, and the learning rate scheduler was used to reduce the learning rate as training progresses.

### 3.4. Model Evaluation

We use the standard mean metric average precision (mAP) to evaluate the model. Intersection over union (IOU) of 0.5 was used as the threshold. Positive means IOU of the predicted bounding box and ground truth is higher than 0.5.

(4) $$\begin{array}{c} AP=\frac{1}{11}\sum \text{precision}\left(\text{recall}\right),\\ mAP=\frac{1}{N}\sum_{i=1}^{N}AP_{i}. \end{array}$$

### 3.5. Model Improvement

To improve the detection accuracy of our model on occlusion players, we have manually collected a large amount of occlusion football players from Tiktok and Youtube videos, manually labeled them, mixed them together with the ISSIA-CNR soccer and soccer player detection datasets to train our model.

To combat multiple spatial scales issues, we use anchor boxes to acquire various scale and aspect ratios of football players, combine feature maps from different convolutional layers, and adopt feature pyramid structures.

We make full use of residual connection to improve the speed of our model, while maintaining high detection accuracy. As the layers go deeper, accuracy will get increased, however, the training parameters grows drastically. The number of convolutional layers is optimized to be 5 in our work.

## 4. Results

### 4.1. Model Performance

Table 2 summarizes the evaluation results of our model on player detection using public datasets ISSIA-CNR and soccer player detection. Our model gives the highest AP score on both datasets (0.915 on the ISSIA-CNR dataset and 0.932 on the soccer player detection dataset) with relatively less training parameters (238 k) and fast inference time (38 numbers of frames per second).

Specifically, for the ISSIA-CNR dataset, our model outperforms both the Faster R-CNN and FootAndBall model. The reason is that our architecture allows our model to capture feature maps with 5 levels of spatial resolution. The residual connections make feature maps from lower level conv layers flow to higher level conv layers easily and also helps the model to converge faster, which leads to shorter training time, fast frames processing, and higher prediction performance. Soccer player detection datasets are created from two different football matches with a wider range of pixels (20–250) than the ISSIA-CNR dataset (63–144 pixels). Our model beats the FootAndBall model, which is probably due to the abovementioned reasons that these residual connections allow all 5 levels of feature maps flow easily to the end before predicting the bounding box and the confidence score. In other words, the special architecture of our model makes it more robust and adapts our model to various football videos.

### 4.2. Effect of the Number of Convolutional Layers on the Model Performance

**Figure 3:**
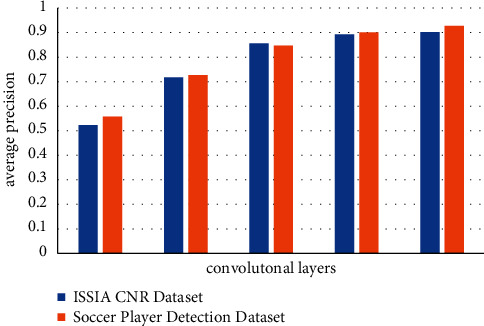
Model performance on two datasets using the feature map extracted from different convolutional layers. Conv1 corresponds to the feature map after the image pass conv1.

To further understand the functions of each block and optimize the model architecture, we extracted feature maps from different convolutional layers and feed them directly to the last 1 × 1 conv and 3x upsample blocks to generate the player confidence map and the bounding box. Then we evaluate the models using the same datasets (the soccer player detection dataset and the ISSIA-CNR dataset) and calculated the average precision. The results are shown in Figure 3.

Figure 3 shows that as convolution layers increase, the average precision of model prediction using both datasets increase. The average precision of the model from conv1 to conv5 on the ISSA-CNR dataset are 0.523, 0.718, 0.856, 0.893, and 0.902, respectively. The percentage enhancement of the average prediction of the model from conv2 to conv5 on the ISSA-CNR dataset compared with conv1 are 37%, 64%, 71%, and 72%, respectively. Similarly, the average precision of the model from conv1 to conv5 on the soccer player detection dataset are 0.557, 0.727, 0.847, 0.901, and 0.927, respectively. The percentage enhancement of the average prediction of the model on the soccer player detection dataset from conv2 to conv5 compared with conv1 are 31%, 52%, 62%, and 66%, respectively. This clearly shows the importance of convolutional layers in object detection. However, as convolutional layers increase, adding more convolutional layers contribute less to the model performance, which is due to the fact that all important feature maps have been extracted. It also indicates that 4–5 layers of convolutional layers is good enough for player object detection tasks.

### 4.3. Effect of the Residual Connection on the Model Performance

**Figure 4:**
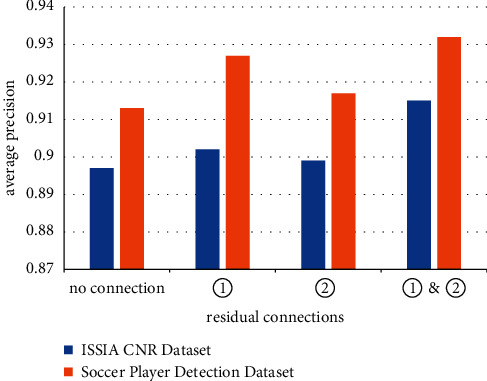
Effect of residual connections on the model performance.

As shown in Figure 4, when there is no residual connection, the model performance is the worst. In fact, the training time is also longer in this case. Adding residual connection 1 increased the model performance much more than adding residual connection 2, which is probably because the feature map after conv3 contains much information from both conv2 and conv1 (comes from residual connection 1), and thus it helps improve the model performance from an average precision of 0.897 to 0.902 (0.55%) for the ISSIA_CNR dataset and from an average precision of 0.913 to 0.927 (1.53%) for the soccer player detection dataset. However, if we only add residual connection 2, the enhancement of the model performance is not so much, which maybe because the spatial resolution of feature maps between conv2 and conv3 have less difference than these between con1 and conv2. Thus, residual connection 2 did not contribute too much information of feature maps with different spatial resolution. When residual 1 and residual 2 are all added, we obtained the best performance, an average precision of 0.915 for the ISSIA_CNR dataset and 0.932 for the soccer player detection dataset.

**Figure 5:**
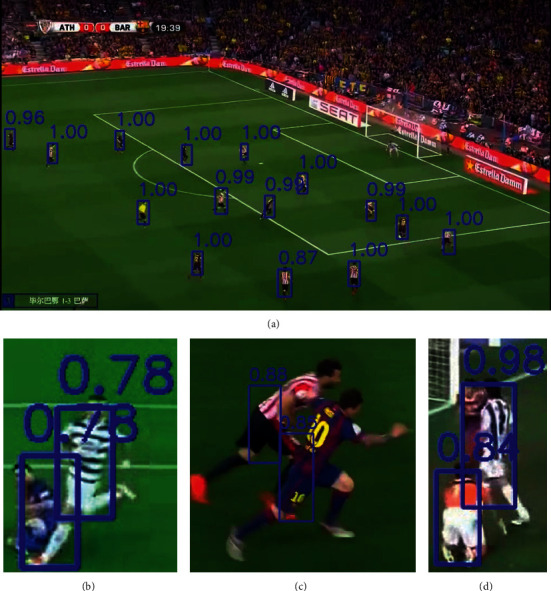
Model performance on (a) a normal football match image for player detection, and on difficult player detection tasks where players are nearby and (b) one player fell on the ground. (c) Plays' legs are overlapped. (d) One player is on his knees.

To further prove the generality of our model, we collect random football match clips from Tiktok and feed it directly into our model for player detection.  Figure 5 shows the model performance on these unseen images from Tiktok. In Figure 5(a), our model can detect all the football players with a confidence score close to 1, which outperforms traditional models. In Figure 5(b), even though the image patch is blurry and one player falls to the ground, our detector still successfully detects both players with a confidence score of 0.78. This is because we use denser grids compared to other popular models, such as YOLO. We scale down the input image size by a factor of 16. In Figure 5(c), two players' bodies are partially overlapped with each other, our model detects both players based on only a part of their bodies and gives a confidence score of 0.88 and 0.85, respectively. In Figure 5(d), one player is on his knees, but our model successfully detect him with a confidence score of 0.84. The high performance of our model on a random football match video clip and on difficult tasks assures the generality and effectiveness of our algorithm.

## 5. Conclusions

In conclusion, we have proposed an efficient deep convolutional neural network-based method to automatically detect football players from video matches directly. Our network was built on the pyramid network with residual connections, with the advantages of single pass fast processing, high robustness, adaptive to all size of images, and suitable for nearly any match videos. Our results shows that it can perform well even on random football match videos obtained from Tiktok, indicating the wide applications of this algorithm. Moreover, our player detection algorithm is faster than the state-of-the-art R-CNN object detector and can be used for real-time football player detection. In the future, we plan to combine transformers with our current deep convolution neural network to not only detect football players, but also predict each player's next action and state.

## Figures and Tables

**Table 1:** Model details in terms of modules, layers and output dimensions.

Module	Layers	Output dimension
conv1	16 filters	
	Max pool 2 d (2 × 2)	[w/2, h/2, 16]
	Residual connection	
conv2	32 filters	
	32 filters	
	Max pool 2 d (2 × 2)	[w/4, h/4, 32]
	Residual connection	
conv3	32 filters	
	32 filters	
	Max pool 2 d (2 × 2)	[w/8, h/8, 32]
conv4	64 filters	
	64 filters	
	Max pool 2 d (2 × 2)	[w/16, h/16, 64]
conv5	64 filters	
	64 filters	
	Max pool 2 d (2 × 2)	[w/32, h/32, 32]
1 × 1 conv1	32 filters	[w/16, h/16, 32]
Player classifier	32 filters	
	2 filters	
	Sigmoid	[w/16, h/16, 1]
Bounding box	32 filters	
	4 filters	[w/16, h/16, 4]

**Table 2:** Comparison of our model with literature models in terms of average precision (AP) of player detection, number of training parameters, and frames per second.

Model	ISSIA-CNR average precision	Soccer player detection average precision	Training parameters (k)	Frames per second	Reference
Faster R-CNN	0.874	0.928	25 600	8	[25]
FootAndBall	0.889	0.834	137	39	[26]
This work	0.915	0.932	238	38	NA

## Data Availability

The dataset is available from the corresponding author upon request.
